# Barriers to optimal diabetes care in Trinidad and Tobago: a health care Professionals’ perspective

**DOI:** 10.1186/s12913-015-1066-y

**Published:** 2015-09-19

**Authors:** Nira Roopnarinesingh, Nancyellen Brennan, Claude Khan, Paul W. Ladenson, Felicia Hill-Briggs, Rita Rastogi Kalyani

**Affiliations:** Division of Endocrinology, Diabetes and Metabolism, Johns Hopkins Medical Institutions, Baltimore, MD USA; Southwest Regional Health Authority, San Fernando, Trinidad and Tobago; Division of General Internal Medicine, Johns Hopkins Medical Institutions, Baltimore, MD USA; Division of Endocrinology, Diabetes and Metabolism, Johns Hopkins University School of Medicine, 1830 East Monument Street, Suite 333, Baltimore, MD 21287 USA

**Keywords:** Barriers, Diabetes, Health care professionals, Caribbean, West Indies

## Abstract

**Background:**

The republic of Trinidad and Tobago (T&T) is a middle income country with a comparatively high prevalence of diabetes mellitus (DM) compared to others in the Caribbean. To date, there have been no studies on health care professionals’ (HCP) perspectives regarding the barriers to achieving optimal care of patients with DM in this country and few previous studies in the Caribbean, yet such perspectives are imperative to develop strategies that reduce the global burden of this disease.

**Methods:**

An electronic invitation was sent to prospective HCP in T&T inviting them to attend a symposium on DM and cardiovascular disease. A total of 198 HCP participants attended of whom approximately 100 participants completed an Audience Response Survey at the completion of the conference. The Audience Response Survey included questions regarding access to resources, need for prevention and education, and coordination of care for to diabetes care in T&T. Responses were analyzed in aggregate.

**Results:**

The 198 HCP participants attending the symposium included mostly nurses (40 %) and physicians (43 %). The most common specialty indicated by the 198 HCP participants was Internal and Family Medicine (28 %), followed by Anesthesiology (7 %), Emergency Medicine (6 %), Endocrinology and Diabetes (5 %) and Cardiology (3 %). Among the ~100 HCP who completed the Audience Response Survey, multiple barriers to achieving optimal care of patients with diabetes were reported such as: limited access to blood testing (75 %), ophthalmological evaluations (96 %), ECGs (69 %), and cardiac stress tests (92 %); inadequate time to screen and evaluate DM complications (95 %); poor access to consultants for referral of difficult cases (77 %); and lack of provider education regarding cardiovascular complications of DM (57 %). HCP agreed that nurses could potentially be considered to have a more active role in the care and prevention of cardiovascular disease and diabetes through leading patient education efforts (98 %), screening patients for complications (91 %), coordinating care efforts (99 %) and educating family members (98 %).

**Conclusions:**

The HCP in our study reported significant barriers to achieving optimal diabetes care in T&T. In the future, such barriers to care will need to be addressed in order to respond to the projected growth of diabetes in developing countries both within the Caribbean and globally.

**Electronic supplementary material:**

The online version of this article (doi:10.1186/s12913-015-1066-y) contains supplementary material, which is available to authorized users.

## Background

In 2010, the global prevalence of diabetes was 6.4 %, or 285 million adults living with diabetes. By 2030, the worldwide prevalence of diabetes is projected to increase to 7.7 %, or 439 million adults [1]. It is anticipated that 69 % of this increase in diabetes prevalence over the next 20 years will be in developing countries. The republic of Trinidad and Tobago (T&T) has a particularly high burden of diabetes compared to other countries. According to the World Health Organization (WHO), the prevalence of diabetes in T&T is expected to more than double from 60,000 to 125,000 between 2000 and 2030 [2]. With an estimated diabetes prevalence of 13 %, T&T was ranked 37th in the world and tenth in North America and the Caribbean according to the International Diabetes Foundation (IDF) in 2013 [3]. In addition, diabetes is costly, with 12 % of global health care expenditures spent on people with diabetes in 2010 [4]. In the mid-1990s, the WHO estimated that diabetes cost $812 million US dollars in the English Caribbean and with an excess health care cost of 329 %, specifically in T&T [5]. Diabetes increases the risk of amputations substantially [6], and is a leading cause of end-stage renal disease and dialysis, blindness and cardiovascular disease and mortality. Consequently, the individual and societal burden of diabetes in T&T is significant and reducing the burden of diabetes could have significant public health benefits.

Furthermore, the majority of patients with diabetes in T&T have likely not achieved optimal glucose goals as recommended by the Caribbean Health Research Council [7]. In 2002, Apparico and colleagues reported that, in North Central Trinidad, only 44.7 % of patients with diabetes were well controlled (hemoglobin A1c < 7 %) [8]. Factors associated with poor glycemic control included older age and long periods between doctors’ visits [9]. However, the reasons for these gaps in diabetes care remained unclear.

Health care professionals who routinely see patients with diabetes offer a valuable perspective on reasons for ongoing gaps in diabetes care. To date, there have been no studies reporting health care professionals’ (HCP) perspectives on the barriers to achieving optimal care of persons with diabetes in the Caribbean, with the exception of Barbados where a previous qualitative study identified barriers at the level of the patient, healthcare professional, and health care system [10]. The perspectives of health care professionals in T&T may uniquely inform the design of future prevention strategies and are imperative to reduce the rising burden of diabetes in this country.

The present study aims to identify barriers to the care of patients with diabetes in T&T from the HCP perspective. We hypothesized that health care professionals in our study would identify multiple barriers to achieving optimal diabetes care in T&T including: limited resource availability, provider knowledge, insufficient time to screen patients, and the need for more specialists and trained nurses.

## Methods

### Study participants

**Fig. 1:**
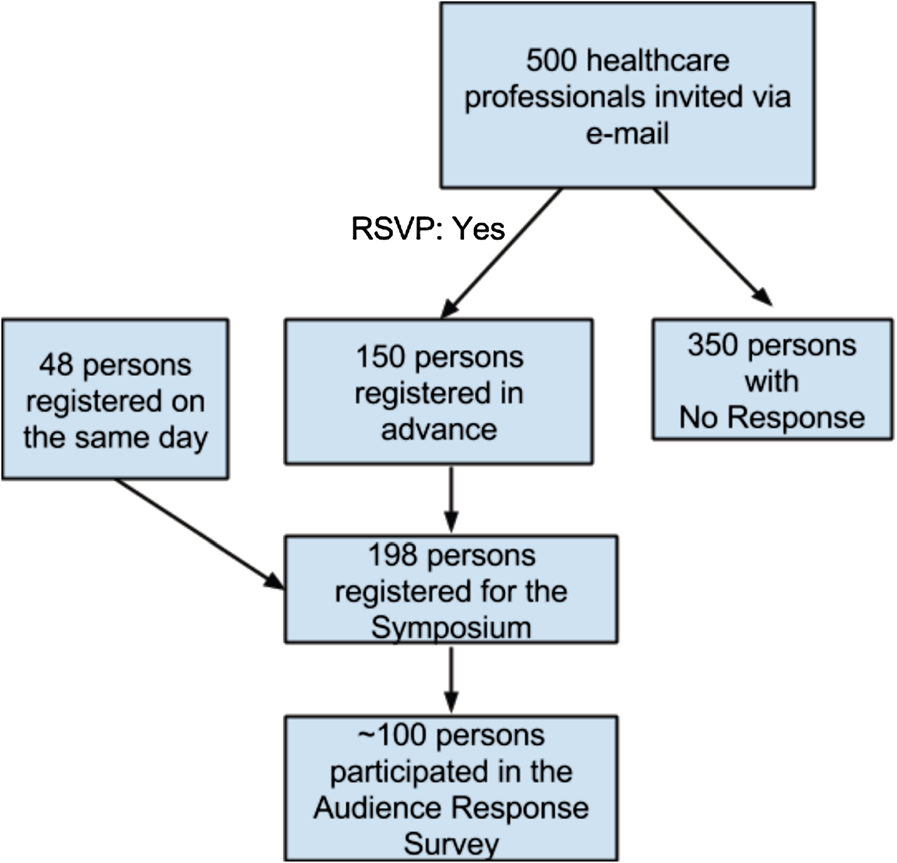
This flow chart demonstrates the selection of HCP Participants who participated in the Audience Response Survey

Study participants attended a continuing education symposium on “Implementing Best Practices for Evaluation and Management of Cardiovascular Disease in Diabetes” sponsored by the Trinidad and Tobago Medical Association (TTMA) and the Trinidad and Tobago Health Science Initiative in September 2011. Electronic (e-mail) invitations to attend the conference were sent to all HCP registered with the TTMA who had provided their email addresses and included approximately 500 health care professionals in Trinidad and Tobago. One hundred and fifty HCPs replied indicating that they would attend. All these persons registered in advance and attended the conference. In addition to those who pre-registered, 48 HCPs registered on the day of the conference and attended the symposium. Thus, there were a total of 198 HCP participants who attended the conference. The registration forms were obtained and the following data were collected: professional degree (MD, MBBS, RN, etc.), region (NWRHA-North West Regional Health Authority, TRHA-Tobago Regional Health Authority, NCRHA- North Central Regional Health Authority, SWRHA - South West Regional Health Authority, etc.), and specialty (e.g., Internal Medicine, Endocrinology, Ophthalmology, etc.). At the completion of the symposium, which included a series of educational lectures on diabetes and cardiovascular diseases, an Audience Response Survey was administered. Approximately 100 health care professionals completed the Audience Response Survey (Fig. 1).

The Johns Hopkins School of Medicine Institutional Review Board approved the study in 2011 as meeting criteria for “exempt” status given that only de-identified data was used, thus, informed consent was not needed. This study was also approved by the T&T Ministry of Health Ethics committee in 2013.

### Audience response survey

Questions for the Audience Response Survey were posed verbally by the presenter and possible response choices displayed on a computer screen; participants could then anonymously choose a response with a handheld electronic response device. Aggregate response rates were then immediately displayed on the screen for all participants to view. Additional file 1 includes the questions administered during the Audience Response Survey. The responses to these questions were recorded as an exact percentage of the total number of participants who participated. The questions were categorized into the following topic areas regarding diabetes care: resource availability, prevention and education, and leadership.

### Statistical analyses

Graphical displays and frequency distributions were constructed for the HCP category and specialty of participants. Descriptive analyses summarized the percentage of participants responding to each individual question regarding barriers to optimal diabetes care.

## Results

**Fig. 2:**
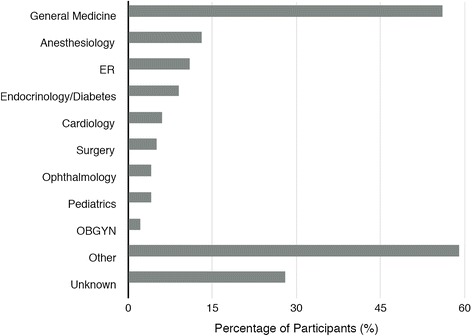
This figure describes the exact percentage of HCP participants, by specialty, who attended the symposium. “General Medicine” includes the specialties of internal medicine, family medicine, gerontology and public health. “Surgery” includes the specialties of thoracic and general surgery. “Other” includes the specialties of dietician, dermatology, orthopedics, psychiatry, radiology, and midwife

The majority of the 198 participants who attended the symposium were physicians (44 %, n = 88) or nurses (40 %, n = 79). Geographically, the most represented regions were from the SWRHA (27.8 %; *n* = 55) and NCRHA (25.8 %; *n* = 51). The most common specialty indicated by HCP participants was Internal and Family Medicine (28 %; *n* = 56), followed by Anesthesiology (6.6 %; *n* = 13), Emergency Medicine (5.6 %; *n* = 11), Endocrinology and Diabetes (4.6 %; *n* = 9) and Cardiology (3 %; *n* = 6) (Fig. 2).

**Fig. 3:**
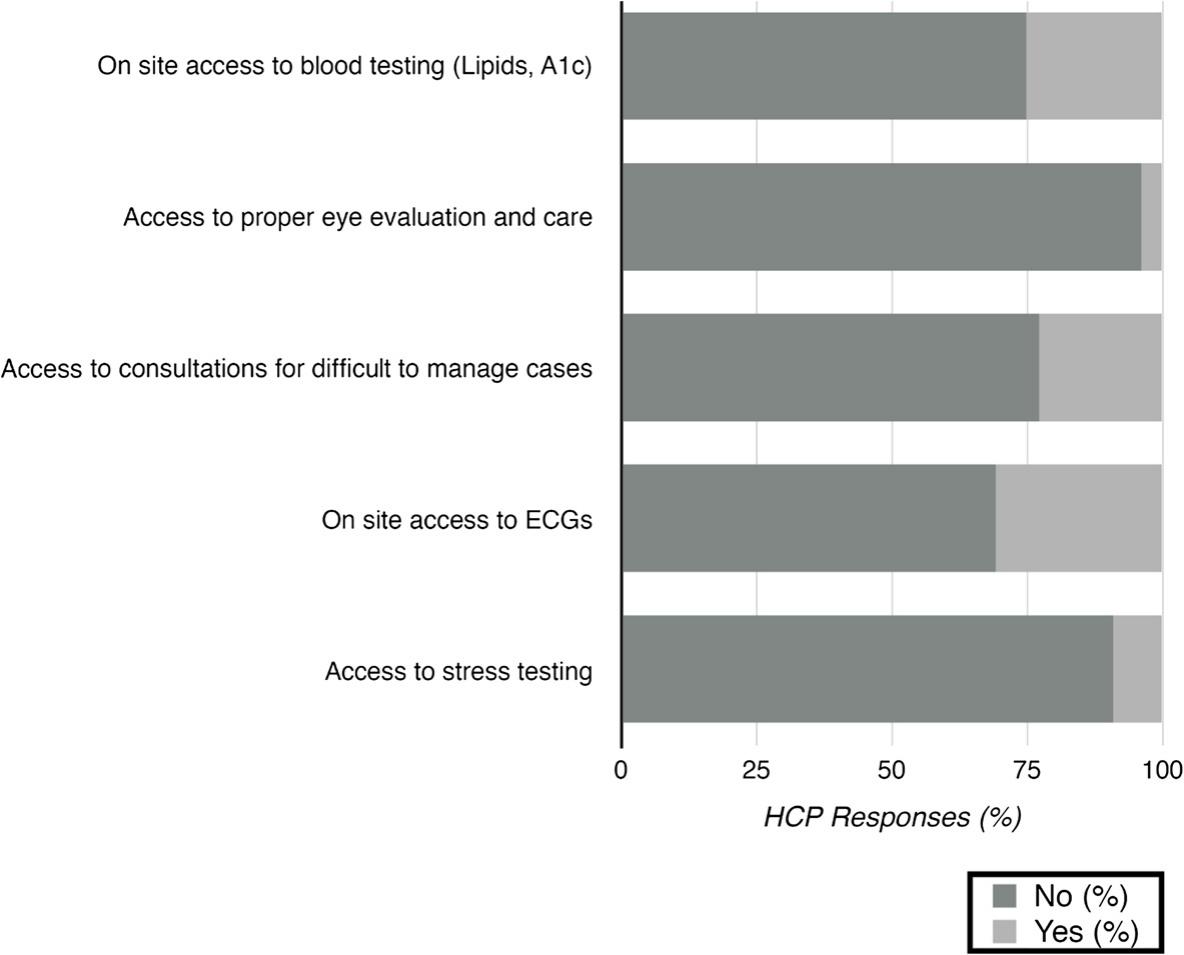
This figure represents the percentage of HCP participants in our study who reported the availability of specific resources for the care of persons with diabetes

Many barriers for providing optimal care for person with diabetes in T&T were indicated by the approximately 100 HCP participants who completed the audience response survey. Participants who did not respond to a particular survey question were coded as missing when analyzing those results. Limited resource availability was identified as a significant barrier (Fig. 3). The majority of HCP participants reported that resources such as access to on-site blood testing (75 %; *n* = 74/99), skilled ophthalmological evaluation and care (96 %; *n* = 99/103), consultations for difficult to manage cases (77 %; *n* = 74/96), on-site ECGs (69 %; *n* = 70/101), and cardiac stress testing (92 %; *n* = 97/106) were inadequate or not being optimally utilized.

**Fig. 4:**
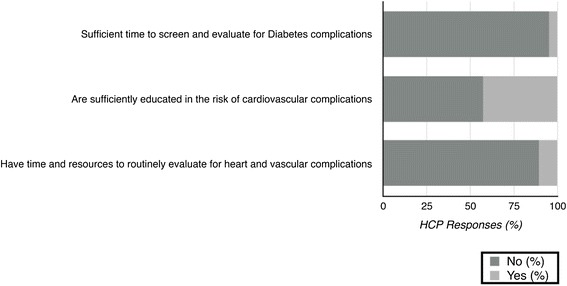
This figure represents the percentage of HCP participants in our study who reported specific barriers in the management of complications when caring for persons with diabetes

Inadequate time to screen for complications and provider education were also identified as additional important barriers to achieving optimal care (Fig. 4). The majority of HCP participants reported that there was neither sufficient time to screen and evaluate diabetic complications (95 %; *n* = 102/107) nor time and resources to routinely evaluate for heart and vascular complications (89 %; *n* = 95/107). Approximately 57 % (*n* = 60/105) reported that health care professionals were not sufficiently educated in the risk of cardiovascular complications.

Ninety percent or more of HCP participants reported that nurses could potentially play a more active role in various aspects of diabetes care such as leading patient education efforts (98 %; *n* = 102/104), educating family members (98 %; *n* = 98/100), screening patients for complications (91 %; *n* = 96/105) and coordinating care efforts (99 %; *n* = 103/104).

All HCP participants reported that prevention of diabetes and cardiovascular complications should be a priority for all HCP. Nearly 90 % or more HCP also indicated that this should also be a priority of the government (88 %; *n* = 92/105) non-governmental organizations, (79 %; *n* = 82/104), popular media (97 %; *n* = 101/104) and schools (88 %; *n* = 96/109).

The most effective media for delivering health care education identified was television (58 %; *n* = 61/105). Eighty percent of HCP participants reported that television together with newspapers, brochures, mailings and posters (20 %; *n* = 21/105) was potentially the best way this information could be delivered.

## Discussion

Our study found that HCP in T&T identified many barriers to caring for persons with diabetes including: limited resources and access to on-site blood testing, ophthalmological evaluations, on-site ECGs and cardiac stress tests, and consultants for complex cases; insufficient provider education; inadequate time to screen for complications; and inefficient coordination of care with trained nurses who could assist in the care of persons with diabetes. To our knowledge, this is the first such study in Trinidad and Tobago to identify barriers perceived by health care professionals when managing persons with diabetes, and one of the largest quantitative studies to date of challenges for diabetes care in the Caribbean.

Studies investigating the perspectives of health care professionals who manage diabetes have been performed in other parts of the world including Ethiopia [11], New Zealand [12], North America [13–17], Europe [18–20] and Asia [21, 22]. These studies demonstrated that HCP report multiple barriers to caring for persons with diabetes such as inadequate funding, limited time to spend with patients, poor access to specialists, ineffective patient and HCP education on diabetes care, and lack of patient motivation to make lifestyle changes. Many of these provider barriers were similarly reported by HCP in our study in T&T.

In Barbados, the only other Caribbean county to have previously reported on HCP perspectives, a qualitative study was performed to identify potential barriers to optimal diabetes care. The provider barriers included the need for continuing medical education, the lack of access to on-site blood tests and clinical equipment, lack of sufficient time per patient visit, lack of sufficient human resources; the need for nurses to take a more active role in patient care rather than performing the job of phlebotomists; and a poorly coordinated approach to diabetes care [10]. The design of the Barbados study and ours were similar in that the HCP participants surveyed were mainly physicians and nurses and the questionnaire was administered verbally. In the Barbados study, patient and system barriers to diabetes care were also queried. Barriers identified included patient denial about their disease and fear of stigma, financial resources to access an appropriate diet and exercise equipment, confusion concerning medication regimens, not valuing free medication, belief in alternative medicines and being unable to change habits. Patients also faced cultural barriers with regards to meals, exercise, appropriate body size, footwear, medication taking, and taking responsibility for one’s health; and difficulty getting time off work to attend clinic. System barriers included lack of access to blood testing, clinic equipment and medication; the lack of human resources in clinics; and an uncoordinated team approach [10]. Compared to the study in Barbados, our study was quantitative and represented a wider variety of subspecialties taking part in the multidisciplinary care for persons with diabetes and additionally demonstrated lack of access to stress tests, ECGs, specialized consultants, and ophthalmologists as barriers to achieving optimal diabetes care in T&T. Our study also identified a need for greater coordination of care with nurses. Patient and system-level barriers to diabetes care in T&T were not the focus of our study, but should be investigated in the future.

Some studies performed in other countries also identified other barriers in treating persons with diabetes such as low income and education level of patients [11, 12, 18–21] and a greater need for HCP to be trained in identifying and addressing patients’ personal barriers and health system [16]. We similarly found that providing training and education was identified as a barrier to optimally managing complications of diabetes in our study.

A few studies additionally describe cultural and language barriers to the care of diabetes related to recent immigration from other countries [12]. In these studies, HCP were not familiar with local peoples’ cultures when placed to work in rural areas, such as an Aboriginal community in Canada [14, 17]. One study done in the United States identified psychosocial problems as a barrier to the care of diabetes [22] and a Belgian study identified paternalistic attitudes of physicians towards patients as another barrier [19]. Such cultural and other barriers could also be explored in future studies in T&T and the Caribbean where many diverse ethnic groups are represented.

The strengths of our study include the diverse study population of both nurses and physicians, who represented various geographical regions in Trinidad and Tobago. The questionnaire included a wide range of topics. We were able to obtain a relatively large study sample size compared to other studies [10] given that the symposium offered a unique opportunity to ascertain HCP perspectives on barriers to achieving optimal diabetes care in T&T, which has not been previously described.

The limitations of the study include possible selection bias; HCP who attended the symposium may be more motivated than other HCP to optimally manage patients with diabetes. Invitations were sent to all members of the Trinidad and Tobago Medical Association which represented the largest database of HCP available at the time. However, this sample may not have included all HCP in the country. The sample size may represent a limitation and larger studies that can further confirm these results are needed in the future. The goals of our study were to provide descriptive information regarding potential barriers to optimal diabetes care in T&T from a HCP perspective. Also, the previous study in Barbados which investigated potential barriers to diabetes care was qualitative in nature and thus findings may not be directly comparable to those of our study. In addition, it is not clear the degree to which participants in our study were aware of the public health sector and its operations, thus, our findings may not necessarily be generalized to all providers in T&T. We had limited information on demographic information for each participant, as well. It is possible that other barriers may have been identified by HCP, beyond those given as options to choose from in the multiple choice format. However, since responses were tabulated in real-time, the multiple choice options allowed us to most efficiently analyze the data for the present study. Future qualitative studies that may help identify other potential barriers to optimal diabetes care are needed. The availability of diabetes education was not queried but also warrants further investigation. Lastly, not all the HCP who attended the symposium in T&T completed the questionnaire at the end of the conference. Given that the audience response survey data was collected in aggregate, we did not have individual-level response data available in order to examine responses by such participant characteristics as age, sex, region, health care profession category, or specialty. However, this should be investigated in future studies. Nonetheless, since the goal of our study was exploratory and includes findings not previously reported in the literature, we propose that such health services research remains of important scientific interest.

## Conclusions

In summary, our study of HCP in Trinidad and Tobago demonstrates many barriers to achieving optimal diabetes care in this country such as resource availability, provider knowledge, inadequate clinic time, and coordination of care. Given the projected rise in diabetes prevalence in the future, our study suggests the need for future studies that can specifically address these ongoing gaps in diabetes care. In addition, such health services research may ultimately inform public health policies for persons with diabetes in Trinidad and Tobago. Studies that additionally investigate patient and system-level barriers to achieving optimal diabetes care are needed. Similar investigations addressing barriers to optimal diabetes care in other countries both within the Caribbean and globally in the future may further facilitate interventions to improve the standard of care for persons with diabetes.

### Availability of data and materials 

Not Applicable.

## Additional file

Additional file 1:
**Audience response survey.** (PDF 124 kb)

## Abbreviations

GP: General practitioner
NGO: Non-Governmental Organization
DM: Diabetes Mellitus
HCP: Healthcare Professionals
CME: Continuous Medical Education
T&T: Trinidad and Tobago
