# Supplementary material for: Barriers to optimal diabetes care in Trinidad and Tobago: a health care Professionals’ perspective
Source: BMC Health Serv Res. 2015 Sep 19;15:396. doi: 10.1186/s12913-015-1066-y (PMC4575420; doi:10.1186/s12913-015-1066-y)
Supplement: Additional file 1: — Audience response survey. (PDF 124 kb) [file 12913_2015_1066_MOESM1_ESM.pdf]

**Additional File 1. Audience Response Survey**

- **Health centers have sufficient resources for evaluation and management of diabetes including (yes/no):**
  - On site access to blood testing (e.g. glucose, lipids, A1C)
  - Sufficient time to screen and evaluate diabetic complications
  - Access to proper eye evaluation and care?
  - Access to consultations for difficult to manage cases?
- **Health care providers managing patients at high risk for cardiovascular disease (yes/no):**
  - Have access to on site access to ECGs
  - Have access to stress testing
  - Have time and resources to routinely evaluate for heart and vascular complications.
- **Optimal use of limited resources could be promoted by (yes/no):**
  - Coordination of all programs by the various government ministries involved in health and education.
  - Allowing each government program to independently pursue its mission

- Coordinating government and NGO health education efforts

- **Health care providers managing patients at high risk for cardiovascular disease (yes/no):**
  - Are sufficiently educated in the risk of cardiovascular complications

- **Prevention of diabetes and cardiovascular complications should be a priority of (yes/no):**
  - The government
  - The Schools
  - Health Care Providers
  - Non-governmental organizations/Associations
  - The popular media
- **The most effective media for health education to the public is:**
  - TV
  - Radio
  - Newspapers, Brochures, Mailings, Posters
  - Internet
  - Structured school curriculum
- **Nurses should play an active role in the care and prevention of cardiovascular disease and diabetes through (yes/no):**
  - Leading patient education efforts
  - Screening patients for complications
  - Coordinating care efforts
  - Educating family members
